# A Lower Bound on the Sinc Function and Its Application

**DOI:** 10.1155/2014/571218

**Published:** 2014-07-08

**Authors:** Yue Hu, Cristinel Mortici

**Affiliations:** ^1^School of Mathematics and Information Science, Henan Polytechnic University, Jiaozuo, Henan 454000, China; ^2^Valahia University of Târgovişte, 18 Unirii Boulevard, 130082 Târgovişte, Romania; ^3^Academy of Romanian Scientists, Splaiul Independenţei 54, 050094 Bucharest, Romania

## Abstract

A lower bound on the sinc function is given. Application for the sequence {*b*_*n*_}_*n*=1_^*∞*^ which related to Carleman inequality is given as well.

## 1. Introduction

The sinc function is defined to be

(1) sinc(x)={sin(x)xx≠0,1x=0.

This function plays a key role in many areas of mathematics and its applications [1–6].

The following result that provides a lower bound for the sinc is well known as Jordan inequality [7]:

(2) $$\begin{array}{c} \text{sinc}\left(x\right)⩾\frac{2}{\pi },\;x\in \left[0,\frac{\pi }{2}\right], \end{array}$$

where equality holds if and only if *x* = *π*/2.

This inequality has been further refined by many authors in the past few years [8–35].

In [36], it was proposed that

(3) $$\begin{array}{c} \text{sinc}\left(x\right)⩾\frac{\pi^{2}-x^{2}}{\pi^{2}+x^{2}},\;x\neq 0. \end{array}$$

We noticed that the lower bound in (3) is the fractional function. Similar result has been reported as follows [1]:

(4) $$\begin{array}{c} \text{sinc}\left(x\right)⩾\frac{53}{53+9x^{2}},\;0⩽x⩽\frac{1}{3}. \end{array}$$

To the best of the authors' knowledge, few results have been obtained on fractional lower bound for the sinc function. It is the first aim of the present paper to establish the following fractional lower bound for the sinc function.

**Theorem 1 .**

For any *x* ∈ [0, *π*], one has

(5) $$\begin{array}{c} sinc\left(x\right)⩾\frac{16\pi^{4}}{{\left(3\pi^{2}+x^{2}\right)}^{2}}-1. \end{array}$$

In [37], Yang proved that for any positive integer *m*, the following Carleman type inequality holds:

(6) $$\begin{array}{c} \overset{\infty }{\underset{n=1}{\sum }}{\left(a_{1}a_{2}\cdots a_{n}\right)}^{1/n}<e\overset{\infty }{\underset{n=1}{\sum }}\left(1-\overset{m}{\underset{k=1}{\sum }}\frac{b_{k}}{{\left(n+1\right)}^{k}}\right)a_{n}, \end{array}$$

whenever *a*_*n*_⩾0, *n* = 1,2, 3,…, with 0 < ∑_*n*=1_^*∞*^ *a*_*n*_ < *∞*, where

(7) $$\begin{array}{c} b_{0}=1,\\ b_{n}=\frac{1}{n}\left(\frac{1}{n+1}-\overset{n-2}{\underset{k=0}{\sum }}\frac{b_{n-1-k}}{k+1}\right),\;\left(n=1,2,\ldots \right). \end{array}$$

From a mathematical point of view, the sequence {*b*_*n*_}_*n*=1_^*∞*^ has very interesting properties. Yang [38] and Gyllenberg and Ping [39] have proved that, for any positive integer *n*,

(8) $$\begin{array}{c} b_{n}>0,\\ b_{n}<\frac{1}{n\left(n+1\right)}. \end{array}$$

In [40], the authors proved that

(9) $$\begin{array}{c} \underset{n\to \infty }{\lim}\frac{b_{n+1}}{b_{n}}=1, \end{array}$$

(10) $$\begin{array}{c} eb_{n}=\overset{1}{\underset{0}{\int }}x^{n-2}h\left(x\right)dx,\;n⩾2, \end{array}$$

where

(11) $$\begin{array}{c} h\left(x\right)=x^{x+1}{\left(1-x\right)}^{1-x}\text{sinc}\left(\pi x\right). \end{array}$$

As an application of Theorem 1, it is the second aim of the present paper to give a better upper bound on the sequence {*b*_*n*_}_*n*=1_^*∞*^.

**Theorem 2 .**

For any positive integer *n*⩾2, one has

(12) $$\begin{array}{c} eb_{n}<\frac{1}{n\left(n+1\right)}-\frac{2-4/\pi }{n\left(n+1\right)\left(n+2\right)}. \end{array}$$

## 2. The Proof of Theorem 1

The proof is not based on (3). We first prove the following result.

**Lemma 3 .**

For any *x* ∈ (*π* − 1/3, *π*], one has

(13) $$\begin{array}{c} sinc\left(x\right)⩾\frac{16\pi^{4}}{{\left(3\pi^{2}+x^{2}\right)}^{2}}-1. \end{array}$$

**Proof**

Set *x* = *π* − *t*, 0 ⩽ *t* < 1/3. Then inequality (13) is equivalent to

(14) $$\begin{array}{c} \pi -t+\sin t⩾\frac{16\pi^{4}\left(\pi -t\right)}{{\left(3\pi^{2}+{\left(\pi -t\right)}^{2}\right)}^{2}}. \end{array}$$

To prove (14) by (4), it is enough to prove that

(15) $$\begin{array}{c} \left(\pi -t\right)+\frac{53t}{53+9t^{2}}⩾\frac{16\pi^{4}\left(\pi -t\right)}{{\left(3\pi^{2}+{\left(\pi -t\right)}^{2}\right)}^{2}}; \end{array}$$

namely,

(16) $$\begin{array}{c} \frac{\left(\pi -t\right)\left(53+9t^{2}\right)+53t}{\left(53+9t^{2}\right)}⩾\frac{16\pi^{4}\left(\pi -t\right)}{{\left(3\pi^{2}+{\left(\pi -t\right)}^{2}\right)}^{2}}. \end{array}$$

Next we prove (16). Let

(17) g(t)=(π−t)(53+9t2)(3π2+(π−t)2)2 +53t(3π2+(π−t)2)2 −16π4(π−t)(53+9t2).

We need only to prove that *g*(*t*)⩾0. Elementary calculations reveal that

(18) g(t)=t2(−9t5+45πt4−144π2t3+(53π+252π3)t2   − 4π2(36π2+53)t+ 636π3).

Noting that, for 0 ⩽ *t* < 1/3, we have

(19) $$\begin{array}{c} -9t^{5}>-\frac{1}{27},\\ -144\pi^{2}t^{3}>-\frac{16\pi^{2}}{3},\\ -4\pi^{2}\left(36\pi^{2}+53\right)t>-\frac{4}{3}\pi^{2}\left(36\pi^{2}+53\right). \end{array}$$

Thus, from (19) and (18), we get

(20) $$\begin{array}{c} g\left(t\right)⩾t^{2}\left(636\pi^{3}-\frac{1}{27}-\frac{16\pi^{2}}{3}-\frac{4\pi^{2}\left(36\pi^{2}+53\right)}{3}\right)⩾0. \end{array}$$

This completes the proof. Now we prove Theorem 1.

**Proof**

By using the power series expansions of sin(*x*) and 16*π* ^4^/(3*π* ^2^+*x* ^2^)^2^, we find that

(21) 1+sinc(x)−16π4(3π2+x2)2 =29+∑n=1∞(−1)n−1un(x2π2)n,

where

(22) $$\begin{array}{c} u_{n}=\frac{16\left(n+1\right)}{3^{n+2}}-\frac{\pi^{2n}}{\left(2n+1\right)!}. \end{array}$$

Set *x* ^2^/*π* ^2^ = *t*, 0 ⩽ *t* ⩽ 1. Consider the function *f*(*t*) defined by

(23) $$\begin{array}{c} f\left(t\right)=\frac{2}{9}+\overset{\infty }{\underset{n=1}{\sum }}{\left(-1\right)}^{n-1}u_{n}t^{n}. \end{array}$$

From (21), we get *f*(0) = 2/9 and *f*(1) = 0.  Lemma 3 implies

(24) $$\begin{array}{c} f\left(t\right)⩾0,\;t_{0}<t⩽1, \end{array}$$

where

(25) $$\begin{array}{c} t_{0}={\left(1-\frac{1}{3\pi }\right)}^{2}\approx 0.79\cdots . \end{array}$$

Elementary calculations reveal that for *n*⩾4,

(26) $$\begin{array}{c} \frac{16\left(2n+1\right)}{3^{3}}>\frac{{\left(3\pi^{2}\right)}^{n}}{\left(2n+1\right)!}. \end{array}$$

Hence, for *n*⩾4, we have

(27) $$\begin{array}{c} u_{n}>0,\\ u_{n}-u_{n+1}=\frac{16\left(2n+1\right)}{3^{n+3}}-\frac{\pi^{2n}}{\left(2n+1\right)!}+\frac{\pi^{2n+2}}{\left(2n+3\right)!}>0. \end{array}$$

Therefore,

(28) $$\begin{array}{c} f\left(t\right)⩾\frac{2}{9}+\overset{6}{\underset{n=1}{\sum }}{\left(-1\right)}^{n-1}u_{n}t^{n}. \end{array}$$

If we set

(29) $$\begin{array}{c} g\left(t\right)=\frac{2}{9}+\overset{6}{\underset{n=1}{\sum }}{\left(-1\right)}^{n-1}u_{n}t^{n}, \end{array}$$

then we have

(30) $$\begin{array}{c} g\left(0\right)=\frac{2}{9}>0,\;\;g\left(1\right)<f\left(1\right)=0. \end{array}$$

The intermediate value theorem implies that there must be at least one root *c* with (0,1) such that *g*(*c*) = 0. Using Maple, we find that on the open interval (0,1) the equation *g*(*t*) = 0 has a unique real root *t*_1_ ≈ 0.89⋯.

Hence, from (28) we get

(31) $$\begin{array}{c} f\left(t\right)⩾0,\;t\in \left[0,t_{1}\right]. \end{array}$$

By (21), (24), and (31), Theorem 1 follows.

## 3. The Proof of Theorem 2

First, we need an auxiliary result.

**Lemma 4 .**

For any *x* ∈ [0,1/2], one has

(32) $$\begin{array}{c} {sinc}^{2}\left(\pi x\right)⩾\frac{1-2x+x^{2}}{1-x+x^{2}}. \end{array}$$

**Proof**

By letting *x* = 1/2 − *t*/2*π*, 0 ⩽ *t* ⩽ *π*, the requested inequality can be equivalently written as

(33) $$\begin{array}{c} \cos t⩾\frac{t^{2}}{2}+\frac{8\pi^{4}}{t^{2}+3\pi^{2}}-\frac{5\pi^{2}+2}{2}, \end{array}$$

so it suffices to show that the function

(34) $$\begin{array}{c} G\left(t\right)=\cos t-\frac{t^{2}}{2}-\frac{8\pi^{4}}{t^{2}+3\pi^{2}}+\frac{5\pi^{2}+2}{2} \end{array}$$

is negative on 0 ⩽ *t* ⩽ *π*. Theorem 1 implies

(35) $$\begin{array}{c} G^{\prime }\left(t\right)<0. \end{array}$$

Hence,

(36) $$\begin{array}{c} G\left(t\right)⩾G\left(\pi \right)=0. \end{array}$$

The required inequality follows. Now we prove Theorem 2.

**Proof**

Let

(37) H(x)={xx(1−x)−xsinc(πx),0<x<11x=0,1.

We first consider the case 0 ⩽ *x* ⩽ 1/2.

Taking the natural log gives

(38) $$\begin{array}{c} \ln H\left(x\right)=\left(x-1\right)\ln x-x\ln\left(1-x\right)+\ln\;\sin\pi x-\ln\pi . \end{array}$$

Taking the second derivative of both sides of (38), we have

(39) $$\begin{array}{c} \frac{HH^{\prime \prime }-H^{\prime 2}}{H^{2}}=\frac{x^{2}-x+1}{x^{2}{\left(1-x\right)}^{2}}-\pi^{2}{\text{csc}}^{2}\left(\pi x\right). \end{array}$$

By Lemma 4, it follows that

(40) $$\begin{array}{c} \frac{HH^{\prime \prime }-H^{\prime 2}}{H^{2}}>0. \end{array}$$

Thus,

(41) $$\begin{array}{c} H^{\prime \prime }>0, \end{array}$$

and therefore for 0 ⩽ *x* ⩽ 1/2, we have

(42) H(x)⩽(1−2x)H(0)+2xH(12)=(2−4π)(−x)+1.

For the case 1/2 < *x* ⩽ 1, since *H*(1/2) = 2/*π*, *H*(1) = 1, and *H* is concave up, it follows that

(43) H(x)⩽2(1−x)H(12)+(2x−1)H(1)=(2−4π)(x−1)+1.

Using (10) from (42) and (43), we have

(44) ebn=∫01xn−2h(x)dx=∫01H(x)(xn−1−xn)dx⩽∫01/2[1−(2−4π)x](xn−1−xn)dx +∫1/21[1+(2−4π)(x−1)](xn−1−xn)dx=1n(n+1)−(2−4π)2n+2−n−4n(n+1)(n+2)2n+1<1n(n+1)−2−4/πn(n+1)(n+2).

This proves Theorem 2.
